# Energy-Efficient Time Synchronization Based on Nonlinear Clock Skew Tracking for Underwater Acoustic Networks

**DOI:** 10.3390/s21155018

**Published:** 2021-07-23

**Authors:** Di Liu, Min Zhu, Dong Li, Xiaofang Fang, Yanbo Wu

**Affiliations:** 1 Ocean Acoustic Technology Center, Institute of Acoustics, Chinese Academy of Sciences, Beijing 100190, China; liudi161@mails.ucas.ac.cn (D.L.); lidong16@mails.ucas.ac.cn (D.L.); fangxiaofang@mail.ioa.ac.cn (X.F.); or wuyanbo@mail.ioa.ac.cn (Y.W.); 2 University of Chinese Academy of Sciences, Beijing 100049, China; 3 Beijing Engineering Technology Research Center of Ocean Acoustic Equipment, Beijing 100190, China; 4 State Key Laboratory of Acoustics, Institute of Acoustics, Chinese Academy of Sciences, Beijing 100190, China

**Keywords:** underwater acoustic networks, time synchronization, nonlinear clock skew

## Abstract

Time synchronization plays an important role in the scheduling and position technologies of sensor nodes in underwater acoustic networks (UANs). The time synchronization (TS) algorithms face challenges such as high requirements of energy efficiency, the estimation accuracy of the time-varying clock skew and the suppression of the impulsive noise. To achieve accurate time synchronization for UANs, an energy-efficient TS method based on nonlinear clock skew tracking (NCST) is proposed. First, based on the sea trial temperature data and the crystal oscillators’ temperature–frequency characteristics, a nonlinear model is established to characterize the dynamic of clock skews. Second, a single-way communication scheme based on a receiver-only (RO) paradigm is used in the NCST-TS to save limited energy. Meanwhile, impulsive noises are considered during the communication process and the Gaussian mixture model (GMM) is employed to fit receiving timestamp errors caused by non-Gaussian noise. To combat the nonlinear and non-Gaussian problem, the particle filter (PF)-based algorithm is used to track the time-varying clock state and an accurate posterior probability density function under the GMM error model is also given in PF. The simulation results show that under the GMM error model, the accumulative Root Mean Square Errors (RMSE) of NCST-TS can be reduced from 10^−4^ s to 10^−5^ s compared with existing protocols. It also outperforms the other TS algorithms in the aspect of energy efficiency.

## 1. Introduction

Underwater acoustic networks (UANs) are important scientific observation platforms for internal observation, information interaction and sensing detection [1,2]. Time synchronization (TS) is an indispensable part of UANs [3,4]. Most of the UANs’ applications depend on TS services. For example, data collection of distributed sensor nodes requires the global time and the TS information is important to the underwater localization systems (especially for the long baseline positioning system) and the communication networking protocols [5,6,7,8] to avoid transmission collisions, such as the Time Division Multiple Access (TDMA) protocol. Moreover, for seismic observation and forecasting, a more accurate time is needed for ocean bottom seismographs.

There are mainly two kinds of methods for sensor nodes to obtain the reference time in UANs. The first is based on the individual atomic clocks, which are more precise than the crystal oscillator clocks but consume a lot of energy. Specifically, the lifetime of a sensor node equipped with an atomic clock is about one-fourth of that equipped with a crystal oscillator [9]. Besides, the atomic clocks also lead to error accumulations in the long term. The second kind is based on the underwater acoustic communication where the time error can be measured by the sensor nodes with the TS algorithms, and it has the advantages of low energy consumption and error compensation. In this paper, we focus on the content of TS algorithms.

There are many TS algorithms proposed for terrestrial wireless sensor networks (WSNs) [10,11,12,13,14,15], and Rhee et al. [16] gives an in-depth analysis and describes the methods of estimating clock parameters of most existing clock synchronization algorithms for WSNs. However, due to the characteristics of the UANs, these algorithms cannot be directly applied [17]. The design of the underwater TS algorithm faces several challenges. First, the underwater propagation delay is not negligible in the UANs due to the low propagation speed of acoustic signals (roughly 1500 m/s in water). Second, underwater sensor nodes are usually powered with batteries [18] and to refresh the batteries, the recovery and deployment of the underwater nodes are expensive [19]. The lack of flexibility imposes a high requirement in the energy efficiency of UANs. As a result, the crystal oscillators with low energy consumption are widely used in the sensor nodes. However, they suffer from temperature variation and thus the frequency is time varying, leading to nonlinear clock drift [20]. Additionally, the signal transmission, which consumes the greatest percentage of the energy compared with the other working stages of the modem, is required to be reduced as much as possible. Third, for the sensor nodes in the UANs, the impulsive noises in underwater environments [21] deteriorate the accuracy of the receiving time stamps. According to the experiments, these complex noises are non-Gaussian, which enhances the TS difficulties.

Several TS algorithms have already been proposed for the UANs [22,23,24,25,26,27,28,29,30]. The algorithm in Cario et al. [22] presented the implementation of an acoustic synchronization and ranging system to enable the control and cooperation of multiple Unmanned Underwater Vehicles (UUVs). It used acoustic transmissions and Chip Scale Atomic Clocks (CSACs) in the SeaModem to overcome the clock drift of real-time clocks (RTS), thus accurate one-way ranging estimation during long-term missions was achieved. TSHL [23] was first put forward for UANs, where the clock skew was estimated by linear regression over multiple one-way reference packet exchanges. In the Tri-message [24], the intervals of reference messages were increased to improve the accuracy and the time-stamp number was decreased to 3. D-sync [25] and NU-Sync [26] leveraged the Doppler shift caused by the relative motion of nodes to achieve synchronization. However, TSHL, Tri-message, D-Sync and NU-Sync assumed constant clock skew during the packet exchanges. Therefore, they all suffer great deteriorations if the initial skew is large.

In the DA-Sync [27] algorithm, the effect of the clock skew during the process of estimating the Doppler scale factor from the physical layer was considered. The APE-Sync algorithm [28] also took the time-varying clock skew into account. It used DE-Sync [29] to collect data that included time stamps of nodes and the estimation of the Doppler scale factor. Then, the data was fed into the Kalman filter to track the variable clock skew periodically. In Yang et al. [30], a new skew estimation model was proposed based on a hybrid approach to characterize the dynamics of the clock skews. The Interactive Multi-Model (IMM) Kalman filter was employed to estimate the time-varying clock state vector. However, the DA-Sync, APE-Sync and ACSE-IMM [30] used the linear models to describe the variation in clock skew which may deteriorate the accuracy of TS [31]. Furthermore, the assumption of the white Gaussian noise model during the multiple two-way communication processes was not accurate enough for the real underwater environments.

The problems of nonlinear variations in the clock skew and non-Gaussian noises cannot be ignored when the sensor nodes work for a long time under the sea. The Kalman filter is the optimal linear filter only when the input entering noise is Gaussian, which is not relevant to this paper. The particle filter-based algorithms are sequential Monte Carlo methods that use finite and large sets of particles to approximate a required probability density, which is suitable for the nonlinear and non-Gaussian problems. To the best of our knowledge, the PF-based TS algorithm has not yet been researched.

In this work, we propose an energy-efficient TS based on nonlinear clock skew tracking (NCST-TS), which aims to overcome the time-varying nonlinear clock skew issue for energy-constrained UANs. The enhancement lies in the following aspects.

- First, to characterize time-varying clock skews, a nonlinear model based on the temperature data collected from sea trials and the crystal oscillators’ temperature–frequency characteristics is established. It compensates for the estimation error introduced by clock skews and increases the TS accuracy.
- Second, based on a receive-only (RO) paradigm, a single-way communication scheme is used to reduce the energy consumption of UANs. By receiving the periodical broadcast signals from the reference node, any sensor node in the communication range can measure the time of arrival (TOA) of the received packets and obtain a series of observation equations that are used to calibrate the clock parameters. The impulsive noises are considered during communication processes and the Gaussian Mixture Model (GMM) is adopted to fit the noise in this paper.
- Last, to solve the nonlinear and non-Gaussian problems, an improved particle filter (PF) algorithm is employed. Moreover, the particles’ weights are revised under the GMM noise model and thus, accurate clock parameters can be estimated.

The simulations demonstrate that the tracking results of the NCST-TS algorithm under the Gaussian and the GMM noise model are better than those of the existing TS algorithms. Under the GMM noise model, the Root Mean Square Errors (RMSE) of NCTS-TS can be reduced from 10^−4^ s to 10^−5^ s. NCST-TS also outperforms existing schemes in energy efficiency.

The rest of the paper is organized as follows: In Section 2, the description of NCST-TS is provided. In Section 3, we show the simulation results that compare NCST-TS and the other TS algorithms. Finally, the conclusions are given in Section 4.

## 2. Algorithm Description

### 2.1. System Model

**Figure 1:**
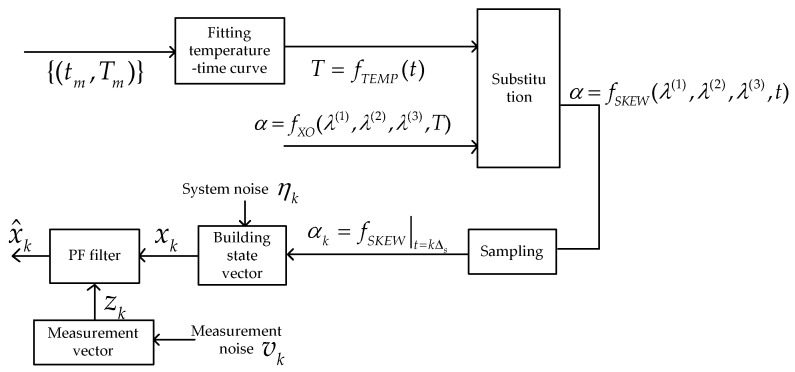
The diagram of the system model.

The model of nonlinear clock skew and the tracking process of the clock skew are shown in Figure 1. It mainly consists of two parts. The first one is the nonlinear clock skew model. The second is the revised PF filter which is used to track the variation in the clock skew.

#### 2.1.1. Nonlinear Clock Skew Model

Attributed to the low-power consumption characteristic of crystal oscillators, they are widely used in sensor nodes to generate a local clock. However, the output frequency of crystal oscillators is temperature sensitive, leading to a nonlinear variation in clock skews [20,31]. Linear models used in existing TS algorithms [28,29,30] are bounded to eliminate the precision of TS accuracy. To achieve an accurate TS, we proposed a nonlinear model to describe the dynamics of clock skews.

Generally, the temperature is constantly changing in a day. Based on the data set of time t and temperature $\left\{t_{m},T_{m}|1\leq m\leq M,\;M\geq 3\right\}$, the function $f_{TEMP}$ is used to express the variation in T according to time t:

(1) $$T=f_{TEMP}\left(t\right).\;$$

On the other hand, the clock skew of the sensor node depends on the shape of the crystal and capacitance and other peripheral equipment. It is influenced by T to a great extent. Function $f_{XO}$ illustrates this relationship, as shown in Equation (2), in which the frequency–temperature characteristics of the quartz crystal is expressed as a third-degree polynomial [32]

(2) $$\begin{array}{c} \alpha =f_{XO}\left(\lambda^{\left(1\right)},\;\lambda^{\left(2\right)},\lambda^{\left(3\right)},T\right)=\overset{3}{\underset{n=1}{\sum }}\lambda^{\left(n\right)}{\left(T-T_{ref}\right)}^{n} \end{array},$$

where $T_{ref}$ is the reference temperature, and $\lambda^{\left(n\right)}\;\left(n=1,2,3\right)$ is the *n*-order frequency–temperature-fitting model parameters of the quartz crystal.

Through formula transformation, the variation in the clock skew according to time in a day can also be obtained. We assume the sensor nodes operate at an identical sampling to interval $\Delta_{s}$, and the discrete form of the clock skew can be written as

(3) $$\alpha_{k}={\overset{3}{\underset{n=1}{\sum }}\lambda^{\left(n\right)}{\left(f_{TEMP}\left(t\right)-T_{ref}\right)}^{n}|}_{t=k\Delta_{s}}={f_{SKEW}\left(\lambda^{\left(1\right)},\;\lambda^{\left(2\right)},\lambda^{\left(3\right)},t\right)|}_{t=k\Delta_{s}}.$$

The recursive form of the nonlinear model of the clock skew can be obtained, which is represented as

(4) $$\alpha_{k}=\alpha_{k-1}+\overset{3}{\underset{n=1}{\sum }}\left(\lambda^{\left(n\right)}{\left(f_{TEMP}\left(k\right)-T_{ref}\right)}^{n}-\lambda^{\left(n\right)}{\left(f_{TEMP}\left(k-1\right)-T_{ref}\right)}^{n}\right).$$

We can define the state vector as

(5) $$x_{k}=\alpha_{k}+\eta_{k},$$

where $\eta_{k}$ denotes the system noise of the clock skew at step k and it represents Gaussian distributions.

#### 2.1.2. Single-Way Communication Scheme under the GMM Noise Model

**Figure 2:**
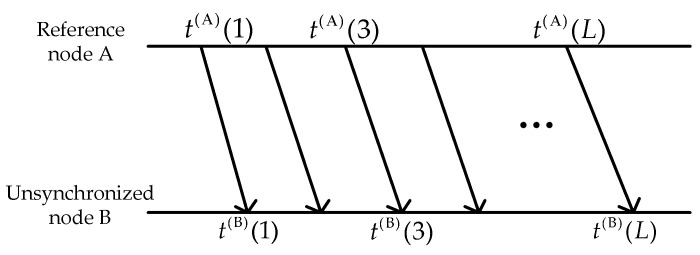
Single-way communication scheme.

The single-way communication scheme in detail is illustrated in Figure 2. Reference node A has the global time. It starts the TS process by sending messages periodically to other sensor nodes, containing its transmission time stamps $t^{\left(A\right)}$. We assume that node A waits for Δ seconds between two successive transmissions. Unsynchronized nodes in their communication range timestamp the local time $t^{\left(B\right)}$ at which it receives the messages from node A. The local time [33] of node B can be written as

(6) $$t_{k}^{\left(B\right)}=\alpha_{k}^{\left(\mathrm{AB}\right)}\times \left(t_{k}^{\left(A\right)}+d^{\left(\mathrm{AB}\right)}+X_{k}^{\left(\mathrm{AB}\right)}\right)+\beta^{\left(\mathrm{AB}\right)}+v_{k}^{\left(B\right)},\;$$

where $\alpha^{\left(\mathrm{AB}\right)}$ and $\beta^{\left(\mathrm{AB}\right)}$ denote the nonlinear time-varying clock skew and the clock offset between two clocks, respectively, at step *k*, and d=D/c is the known part of the propagation delay, which is assumed as a constant value. D represents the distance between two nodes and c is the speed of sound underwater. We assume c=1500 m/s. The *i.i.d.* zero mean Gaussian random variable $X^{\left(\mathrm{AB}\right)}$ denotes the random delay and $v^{\left(B\right)}$ is measurement noise in UANs.

As for the underwater acoustic channel, underwater noises include environment noise, radiation noise and self-noise of targets, etc. When there is an impulsive noise in actual noise, it causes receiving errors of timestamps, which will deteriorate the accuracy of TS. In this paper, the GMM [34] is used to fit receiving timestamp errors caused by non-Gaussian noise. The probability density distribution of measurement noise $v^{\left(B\right)}$ is

(7) $$p\left(v^{\left(B\right)}|\Theta \right)=\overset{I}{\underset{i=1}{\sum }}\phi_{i}N\left(v^{\left(B\right)}|\mu_{i},\sigma_{i}^{2}\right),$$

where Θ={$\phi_{i},\mu_{i},\sigma_{i}^{2}$} is a parameter set of GMM, *M* is the number of components, $N\left(\mu_{i},\sigma_{i}^{2}\right)$ is the probability density function of the i-th Gaussian component with the mean value at $\mu_{i}$ and variance value at $\sigma_{i}^{2}$ and $\phi_{i}$ is the weight value of the i-th Gaussian component which is satisfied when $\overset{I}{\underset{i=1}{\sum }}\phi_{i}=1$.

After several rounds of message exchanges, node B divides the received timestamps into several groups. Four timestamps $t_{2k-1}^{\left(A\right)},\;\;t_{2k-1}^{\left(B\right)},\;t_{2k}^{\left(A\right)},\;\;t_{2k}^{\left(B\right)}$ consist of a group. Then, we can produce Equation (8):

(8) $$t_{2k}^{\left(B\right)}-t_{2k-1}^{\left(B\right)}=\alpha^{\left(\mathrm{AB}\right)}\left(k\right)\times \left(t_{2k}^{\left(A\right)}-t_{2k-1}^{\left(A\right)}+X_{2k}^{\left(\mathrm{AB}\right)}-X_{2k-1}^{\left(\mathrm{AB}\right)}\right)+\left(v_{2k}^{\left(B\right)}-v_{2k-1}^{\left(B\right)}\right).$$

More specifically, under the assumption that $t_{2k}^{\left(A\right)}-t_{2k-1}^{\left(A\right)}\gg \left(X_{2k}^{\left(\mathrm{AB}\right)}-X_{2k-1}^{\left(\mathrm{AB}\right)}\right)$, which is verified in the considered underwater case, by stacking the observations in vector form, the measurement equation can be presented as $z_{k}=Hx_{k}+v_{k}^{\left(B\right)},$ where $z_{k}$, H,$\;v_{k}^{\left(B\right)}$ can be obtained as

(9) $$z_{k}=t_{2k}^{\left(B\right)}-t_{2k-1}^{\left(B\right)},$$

(10) $$H=t_{2k}^{\left(A\right)}-t_{2k-1}^{\left(A\right)},$$

(11) $$v_{k}^{\left(B\right)}=v_{2k}^{\left(B\right)}-v_{2k-1}^{\left(B\right)}.$$

#### 2.1.3. Calibration of the Clock offset

**Figure 3:**
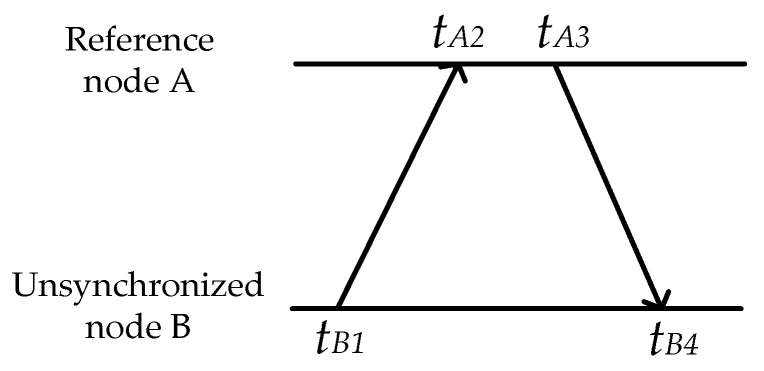
Message exchange process of clock offset calibration.

The process of clock offset calibration is similar to the classical two-way synchronization exchange as shown in TPSN [11] and Figure 3. The unsynchronized node B sends messages to reference node A at time $t_{B1}$, the skew-corrected local timestamp. Node A receives the message at time $t_{A2}$ and responses to node B at time $t_{A3}$. Node B receives the response at time $t_{B4}$. Then, node B can correct the clock offset through

(12) $$offset_{B}=\frac{\left[\left(t_{A2}-t_{B1}\right)-\left(t_{B4}-t_{A3}\right)\right]}{2}.$$

### 2.2. Nonlinear Clock Skew Tracking Based on PF

Because of the nonlinear time-varying clock skews and non-Gaussian distribution of measurement errors, PF algorithms [35] are adopted to track the clock state in NCST-TS. Estimating results of the clock state can be calculated by particles and associated weights. The weights are dependent on the likelihood function $p\left(z_{k}|x_{k}^{\left(n\right)}\right)$, which is revised under the GMM error model in this paper.

**Figure 4:**
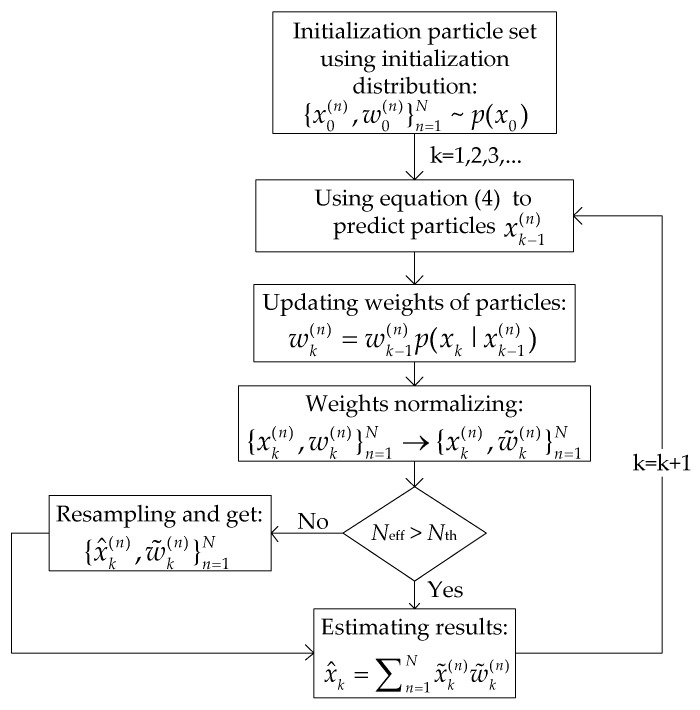
Diagram of tracking nonlinear clock skew using PF under GMM noise model.

The steps of tracking nonlinear clock skew are shown in Figure 4.

Based on the prior probability density $p\left(x_{0}\right)$, initial particles ${\left\{x_{0}^{\left(n\right)}\right\}}_{n=1}^{N}$ and weights ${\left\{w_{0}^{\left(n\right)}\right\}}_{n=1}^{N}$ are generated at k=0. The weight of each particle is $w_{0}^{\left(n\right)}=1/N,1\leq n\leq N$. Particles at *k* step $x_{k}^{\left(n\right)}$ can be calculated from Equation (4) and (5).

In this section, the sampling importance resampling (SIR) filter [36] is considered, thus prior density $p(x_{k}|x_{k-1}^{\left(n\right)})$ is chosen to be the importance density. Under the GMM noise model, the weights of particles are revised and updated as

(13) $$\begin{array}{ll} w_{k}^{\left(n\right)} & =w_{k-1}^{\left(n\right)}p\left(x_{k}|x_{k-1}^{\left(n\right)}\right)\\ & =\overset{M}{\underset{m=1}{\sum }}w_{k-1}^{\left(n\right)}\times \phi_{i}\times \frac{1}{\sqrt{2\pi }\sigma_{i}}e^{-\frac{{\left(z_{k}-H_{k}\left(x_{k-1}^{\left(n\right)}\right)\right)}^{2}}{2\sigma_{i}^{2}}}. \end{array}$$

Then, the weight is normalized as

(14) $${\tilde{w}}_{k}^{\left(n\right)}=\frac{w_{k}^{\left(n\right)}}{\sum_{n=1}^{N}w_{k}^{\left(n\right)}},$$

and an approximation estimation of the posterior probability density function at time *k* can be expressed as

(15) $$p\left(x_{k}|z_{1:k}\right)\approx {\left\{x_{k}^{\left(n\right)},{\tilde{w}}_{k}^{\left(n\right)}\right\}}_{n=1}^{N}.$$

For the particles at time *k*, the resampling method is used to solve the problem of the degeneracy phenomenon in PF algorithms [37]. If the number of effective particles ($N_{eff}$) falls below some threshold ($N_{th}$)

(16) $$N_{eff}\approx \frac{1}{\sum_{n}w_{k}^{\left(n\right)}}<N_{th},$$

a new set of particles $\;{\left\{{\tilde{x}}_{k}^{\left(n\right)}\right\}}_{n=1}^{N}$ is generated by eliminating particles having low-importance weights and by multiplying particles having high-importance weights [36]. We employ the multinomial resampling method in this paper. The weights are now reset to 1/N. After resampling, we have a new set of particles ${\left\{{\tilde{x}}_{k}^{\left(n\right)},1/N\right\}}_{n=1}^{N}$.

Finally, the estimation value at time *k* of the clock skew is

(17) $${\hat{x}}_{k}=\frac{1}{N}\times \overset{N}{\underset{n=1}{\sum }}{\tilde{x}}_{k}{}^{\left(n\right)}.$$

## 3. Performance Evaluation

### 3.1. Simulation Setup

The performance of the proposed TS algorithm NCST-TS was compared with APE-Sync, Tri-Message and the classical PF algorithm. APE-Sync was based on the Kalman filter algorithm. In the Tri-message, the influence of the clock skew during TS processes was not considered. The classical PF algorithm assumed the noise satisfied the Gaussian distribution for simplicity [35]. The parameters used in our simulations are shown in Table 1. Without loss of generality, the average of each data point is obtained by 1000 runs.

### 3.2. Simulation Results and Analysis

#### 3.2.1. Performance of Tracking Results and RMSE Based on the Gaussian Noise Model

Through the observation of the collected experimental data, the Fourier series model is used to describe temperature variation as follows:

(18) $$T\left(t\right)=\overset{Q}{\underset{q=0}{\sum }}a_{q}\cos\left(q\omega_{0}t\right)+b_{q}\sin\left(q\omega_{0}t\right)$$

where $\left\{a_{q}|0\leq q\leq Q\right\}$, $\left\{b_{q}|1\leq q\leq Q\right\}$, $\omega_{0}$ are the fitting coefficients, and *Q* denotes the number of Fourier series. Based on this temperature model, we carried out a series of simulations.

When the noise model satisfies the Gaussian distribution, NCST-TS is the same as the classical PF algorithm. As a result, first, we demonstrate the advantage of the proposed NCST-TS algorithm over the aforementioned APE-Sync and Tri-message algorithms based on the Gaussian noise model.

**Figure 5:**
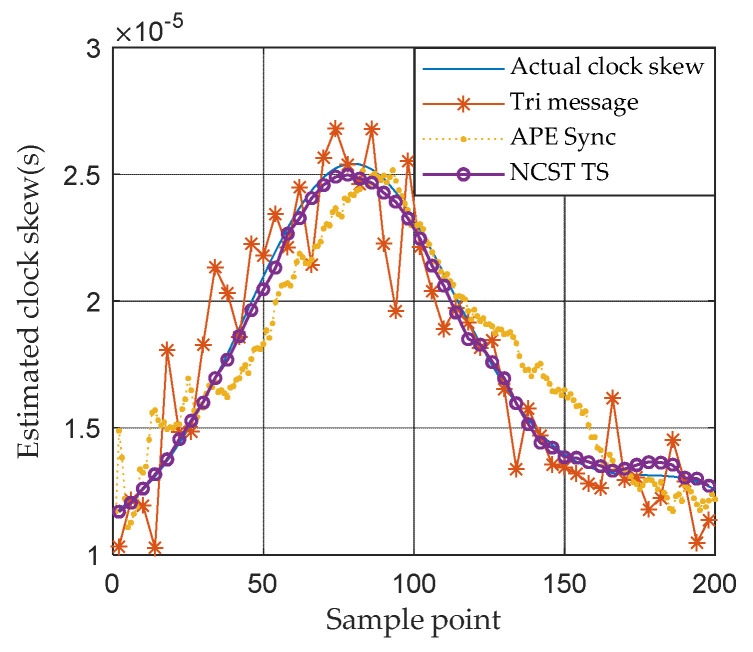
Clock skew tracking results of different TS algorithms based on Gaussian noise model.

**Figure 6:**
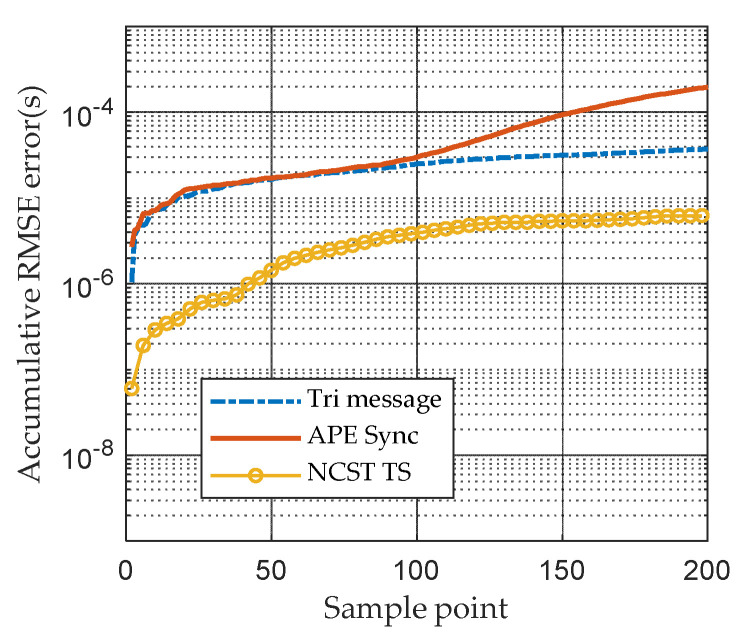
Accumulative RMSE caused by skew of different synchronization algorithms based on Gaussian noise model.

In Figure 5, the clock skew tracking results of three different TS algorithms are compared. In Tri-message, it is assumed the clock skew is invariable during the communication processes, which cannot track the time-varying clock skew well. The APE-Sync algorithm considers the variation in clock skew, fortunately. However, it uses the Kalman filter to track the state of the clock skew which is not suited to the nonlinear model. Compared with APE-Sync, PF-based NCST-TS can handle the nonlinear characteristic of the system well. It outperforms both the Tri-message and APE-Sync. Figure 6 shows the accumulative RMSE of APE-Sync, Tri-message and NCST-TS based on the Gaussian noise model, respectively, in a day. NCST-TS shows the best tracking performance among the three synchronization methods. It is easy to understand that the proposed algorithm has the smallest TS error. At the end of the simulation, the accumulative RMSE of the proposed method is $3.8\times 10^{-6}s$.

#### 3.2.2. Performance on Tracking Results and RMSE Based on the GMM Noise Model

To investigate the performance of different TS algorithms in a more realistic underwater environment, artificial mixed Gaussian errors are added to the received time stamps. The mixed Gaussian errors follow a distribution in Equation (7).

**Figure 7:**
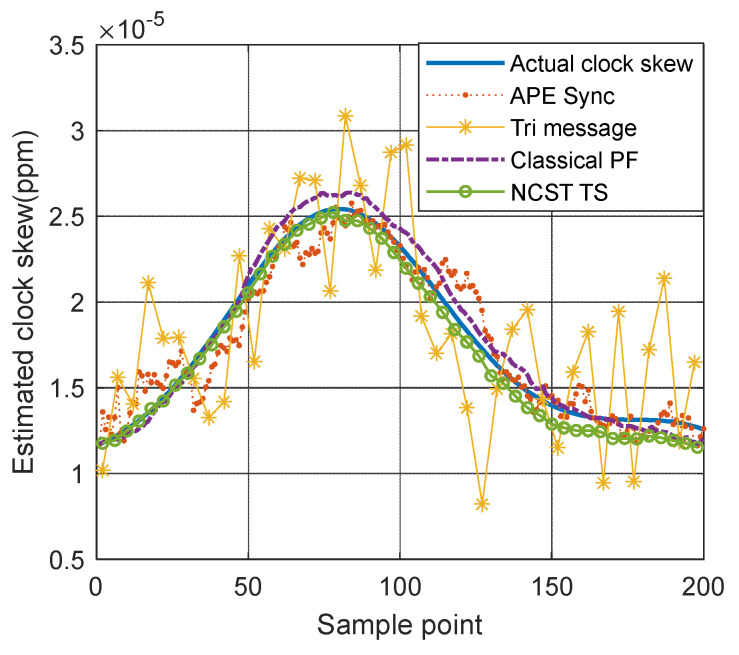
Clock skew tracking results of different TS algorithms based on GMM noise model.

In Figure 7, based on the GMM noise model, the clock skew tracking results of four TS algorithms, including APE-Sync, Tri-message, the classical PF algorithm and NCST-TS, are compared. Clearly, NSCT-TS gives the best estimation results. The Tri-message only uses four time stamps to calibrate clock parameters which are easily influenced by impulsive noise. As shown in Figure 7, at the moment the impulsive noise happens, the estimated errors of clock parameters are obvious. In the Kalman filter-based APE-Sync algorithm, the linear system model and Gaussian measurement noise are assumed. With the GMM measurement noise model and nonlinear clock skew model that are used in this work, APE-Sync also results in a large number of errors in the estimation of clock parameters. NCST-TS is not limited to these conditions. Moreover, considering GMM measurement noise, NCST-TS uses Equation (14) to re-assign the weights of particles. The new particles and weights are used to represent the required posterior density under the Gaussian mixture model. Compared with the classical PF algorithm, the NCST-TS algorithm has a better performance.

**Figure 8:**
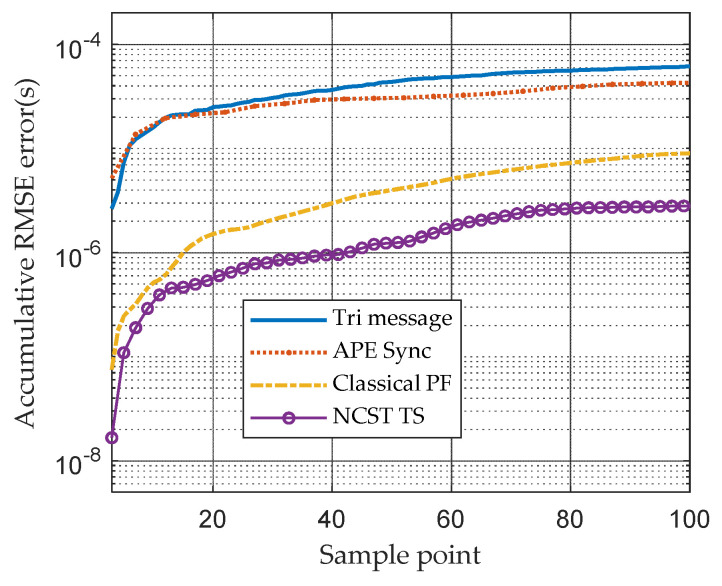
Accumulative RMSE caused by clock skew of different TS algorithms based on GMM noise model.

As shown in Figure 8, the accumulative RMSE of the APE-Sync algorithm and Tri-message TS method increase quickly because of the problem of ignorance of time-varying clock skews. Compared to them, the conditional PF method and NCST-TS have better performance and the NCST-TS algorithm outperforms other TS methods. At the last moment of a day, the accumulative RMSE of the proposed method under the GMM noise model is $3.3\times 10^{-6}s$.

#### 3.2.3. Comparison of Different Algorithms in Terms of Energy Efficiency

**Figure 9:**
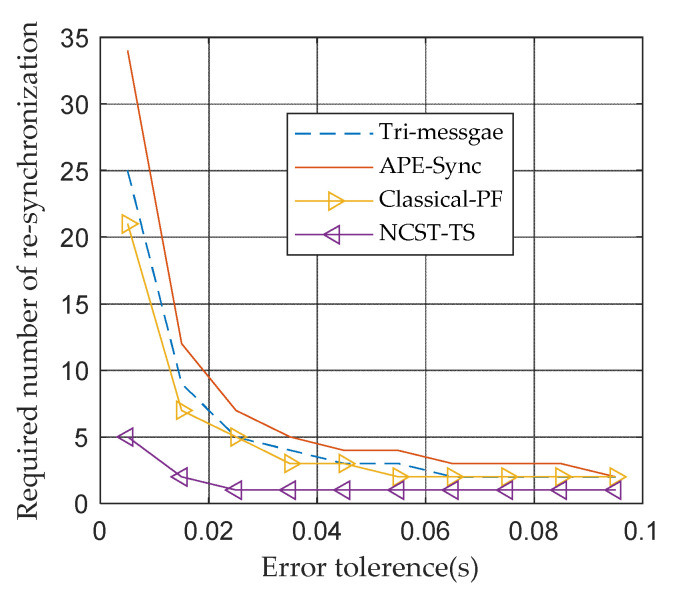
Required number of re-synchronization based on GMM noise model.

Figure 9 compares the required number of re-synchronizations of different synchronization methods under the GMM noise model. The definition is shown in Equation (19) [38],

(19) $$\Omega =\frac{P}{\left(\frac{\hat{\alpha }\varepsilon +\left(\hat{\beta }-\beta \right)}{\alpha -\hat{\alpha }}\right)}$$

where P represents a period of time after TS completes and P is set to 10^5^ s. ε is the tolerance error, α and $\hat{\alpha }$ are the true clock skew and estimated clock skew and  β and $\hat{\beta }$ are the true clock offset and estimated clock offset, respectively. More energy is consumed according to the increase in the synchronization repeating times.

Figure 9 shows that as the value tolerance error increases, Ω will become smaller. The re-synchronization times are dependent upon the accuracy of estimated clock parameters. The NCST-TS algorithm has the highest estimated accuracy among the four synchronization methods. As a result, it needs a minimum number of re-synchronizations and it has the highest energy efficiency, which is suitable for UANs.

#### 3.2.4. Comparison of Different Algorithms in Terms of Time Error after TS

**Figure 10:**
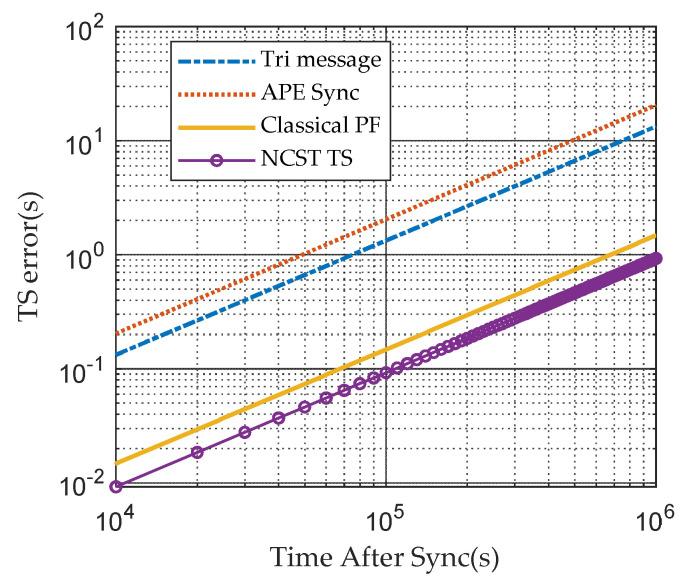
After TS completes, the clock error vs. time after synchronization based on GMM noise model.

The error curves of Tri-message, APE-Sync, the classical PF algorithm and the NCST-TS algorithm under the GMM noise model are depicted in Figure 10. Clearly, the NCST-TS algorithm works much better than APE-Sync, the Tri-message protocol and the classical PF method. The NCST-TS scheme achieves a more precise skew, which reduces TS errors. Tri-message and APE-Sync cannot track the nonlinear model of clock skews, which results in higher TS errors. Compared with the classical PF algorithm, NCST-TS recalculates the particles’ weights under the GMM noise model, which gives a more precise posterior density function. As a result, the NCST-TS algorithm estimates clock parameters well and corrects the shortcomings of other schemes, which is better than the other TS algorithms.

#### 3.2.5. Comparison of Different TS Algorithms in Terms of Consumed Energy

We assume the TS rounds of APE-Sync and NCST-TS are the same for 20 times. The number of time stamps of the sensor node transmitting and receiving, and the energy consumed by APE-Sync and NCST-TS, are shown in Table 2. In our simulations, 20 particles are used.

We assume the transmitting power is 2 W, the receiving power is 0.75 W and the transmission delay and the receiving delay are $t_{s}$, $t_{r}$. and $t_{s}=t_{r}=$0.7 s. The CPU we used is Inter(R) Core(YM) i7-8750H @ 2.2 GHz and the thermal design power (TDP) is 45 W. The energy consumed by NCST-TS and APE-Sync on the computer is 0.333 and 0.158 J, respectively. However, compared with processing energy, the transmission energy is the major consumption aspect. Due to the single-way communication scheme of NCST-TS, it saves 50% energy compared with APE-Sync. Although there are differences in energy consumption on the modem’s DSP platform, the difference will not exceed 10 times, which is acceptable.

In other words, although NCST-TS uses PF to track the variation in the clock skew, it decreases the transmission frequency, which saves most of the energy of the sensor node.

## 4. Conclusions and Future Work

An energy-efficient TS algorithm based on a nonlinear clock skew model for UANs, named NCST-TS, was proposed in this paper. To combat the problem of nonlinear clock drift caused by temperature variation in sensor nodes, a nonlinear model was established to describe the dynamic of clock skews. It is more accurate than the linear model. Then, a single-way communication scheme was used by sensor nodes to synchronize with the reference clock only through receiving reference messages. This scheme conserved a lot of energy for the whole network. Moreover, during the communication process between sensor nodes and the reference node, the GMM noise model was introduced to fit the non-Gaussian errors of the receiving timestamps in underwater environments. Finally, to deal with nonlinear and non-Gaussian problems, sensor nodes tracked the variation in clock states based on an improved PF method. The particles’ weights formula under the GMM noise model were revised. Our simulation results show that compared with existing algorithms, the NCST-TS algorithm could track the clock skew well, and it is a high-precision algorithm with a low message overhead.

Future research will focus on the exploitation of the concepts presented here to support mobile nodes TS and multi-hop TS in large UANs. A network with well-synchronized clock parameters may have sufficient fidelity to perform localization and navigation purposes.

## Figures and Tables

**Table 1:** Simulation parameters.

Parameters	Value
Distance D	1500 m
Speed of sound c	1500 m/s
Maximum skew of α	40 ppm
Maximum offset of β	0.01 s
Interval between transmit messages △	2 s
Number of message *L*	30
Granularity of clock	0.1 μs
random delay *X*	10 μs

**Table 2:** Comparison of different methods under GMM noise model.

Algorithm	Run Time (ms)	Number of Transmitting Time Stamps	Number of Receiving Time Stamps	Energy Consumed by Processing (J)	Energy Consumed by Trans and Revs Activities (J)
NCST-TS	7.4	1	21	0.333	12.425
APE-Sync	3.5	11	11	0.158	21.175

## Data Availability

Not applicable.
